# Comorbidities of cardiovascular disease and cancer in hemophilia patients

**DOI:** 10.1186/s12959-016-0097-x

**Published:** 2016-10-04

**Authors:** Jiaan-Der Wang

**Affiliations:** 1 Center for Rare Disease and Hemophilia, Department of Pediatrics, Taichung Veterans General Hospital, Taichung, Taiwan; 2 School of Medicine, China Medical University, Taichung, Taiwan

**Keywords:** Hemophilia, Cardiovascular disease, Cancer, Clotting factor

## Abstract

As life expectancy greatly increases in persons with hemophilia (PWH), more age-related diseases such as cancer and cardiovascular disease (CVD) emerge among this patient group. The aim of this study was to review the available evidence on the epidemiology of CVD events, and incidence and survival of cancer in PWH. The prevalence of CVD events among PWH seems to be similar to that of the general population. Some known risk factors for the event, including aging, hypertension, and hyperlipidemia, are also associated with its occurrence in PWH. There is no evidence showing occurrence of the event directly to clotting factor concentrate administration. On the other hand, the incidence of non-virus related cancer seems to be higher in PWH than the general population. In addition, PWH with cancer were younger at the time of diagnosis. In regards to hemophilia effect on cancer prognosis, further basic and large-scale prospective studies are urgently needed.

## Background

The challenges in hemophilia care change with time. Life expectancy was increased in persons with hemophilia (PWH) after the introduction of clotting factor concentrates (CFCs) in the 1970s. But complications due to hepatitis C virus (HCV) or human immunodeficiency virus (HIV) contaminated clotting factor products resulted in the main health problem in the 1980s and 1990s [1, 2]. As recombinant CFCs commercialized, life expectancy in PWH nearly approaches the general population [3, 4]. However, clinical physicians are facing new challenges, mostly age-related disease [5, 6]. Older PWH are at risk for developing aging comorbidities, such as cardiovascular disease (CVD) and cancer. Hemophilia is a hereditary bleeding disorder that results from the absence or deficiency of clotting factor. It is plausible that a hypocoagulable state, such as that present in PWH or hemophilia carriers, have a protective effect on thrombus formation, which precipitates CVD events [7, 8]. And, a presumed protection of these inherited bleeding disorders against cancer progression is particularly intriguing [9]. However, to date, there are few cohort studies involving PWH with CVD and cancer, and the risk for these two age-related comorbidities in PWH is often left to physician’s discretion. In addition, rare research is focused on how CFCs affect these two comorbidities. There is considerable interest in the complex interaction between bleeding tendency and age-related disease among PWH. This review aimed to evaluate prevalence of CVD events, and cancer incidence and survival among PWH in comparison with the general population. In addition, the association between CFC use and the two age-related diseases was investigated.

## Review

### Cardiovascular diseases in hemophilia patients

Since hemophilia A and B patients are absence or deficiency of coagulation factors, it is plausible that deficiency of coagulation factor VIII or IX exerts a protective effect on the development of CVD events. However, CVD events are increasingly being reported in the hemophilia populations. It is probably caused by significant increase in the life expectancy of PWH. Several studies have found that the incidence of or mortality due to CVD events in PWH was lower than that in the general population [3, 10–13]. However, some studies have shown that PWH have a similarly high prevalence of atherosclerotic plaques as the general population [14–16]. Moreover, in a systematic review, mortality due to CVD was found to be non-significantly reduced in PWH as compared with the general population [17].

A nationwide analysis conducted to compare CVD events between PWH and general population in Taiwan was reported in 2015 [18]. The data was obtained from the catastrophic illness lists of the National Health Insurance Research Database (NHIRD). Data of the general male population for comparison with the hemophilia group was retrieved from the Longitudinal Health Insurance Database by matching the birth month. The population-based survey showed that the prevalence rate of CVD events including ischemic heart disease, ischemic stroke and peripheral arterial disease is similar between PWH and the general population. However, the result on the prevalence of CVD events between PWH and general population is inconsistent in the literature. In 2011, Biere-Rafi et al. reported that the expected risk of CVD events among PWH is comparable to that in the general population [17]. A study conducted in the Netherlands and the UK corroborates this result [19]. However, several cohort studies on the prevalence of CVD, even all in the United States, are contradictory. In 2005, Kullarni et al. reported that PWH with ischemic heart disease had lower hospital discharge rate than those of an age-matched population without coagulation disorders [10]. Ragni et al. reported that the prevalence of CVD in PWH was similar with that of the general population [20]. Another study conducted by Sharathkumar et al. found that PWH were almost twice as likely to have CVD, compared with individuals without hemophilia [21]. The data on survey of 2560 USA males with hemophilia A patients showed similar findings [22]. There are several possible explanations for the discrepancy among these studies. First, the extent to which age-matched reference data was properly applied may have varied among these studies. Second, the definition of CVD in these studies was different. Some cardiovascular problems including cardiomyopathy, valvular heart disease and rhythm disturbance were defined as CVD events. Finally, the proportion of PWH with severe hemophilia, whose low level of clotting factor has a protective effect on CVD occurrence and may have influenced the results, was diverse and not analyzed separately.

There are also few data on investigation of the risk factors for CVD events among PWH [23, 24]. In addition, few studies have investigated whether CFC use contributes to the development of CVD events in the hemophilia population. In our survey of 1105 PWH collected from Taiwan’s NHIRD between 1997 and 2011, sixty-four arterial thrombotic events, including 32 with ischemic heart disease, 27 with ischemic stroke, and 7 with peripheral arterial occlusive disease, and 5 venous thrombotic events were identified [25]. Less CFC use was found in those PWH with than without CVD events. In addition, PWH who needed replacement therapy had a lower risk of CVD events than those who did not need, with a hazard ratio (95 % CI) of 0.41 (0.21–0.81). The results could be interpreted as the occurrence of CVD events inversely related to the severity of hemophilia. Furthermore, analysis of the case-crossover design showed the amount of CFC use in PWH with CVD events was not increased prior to the event. Based on the result of the nationwide and population-based report, no evidence support CFC administration in PWH is a risk factor for CVD occurrence. Another interesting issue in terms of CVD events not well addressed now is if there is any difference between PWH who are on purely recombinant clotting factors and plasma-derived clotting factors. Purity of clotting factor VIII or factor IX prothrombin complex usage may contribute to the risk of CVD events.

Obesity has been reported to be associated with CVD events in the general population [26, 27]. In addition, it is an emerging issue contributing to chronic disease and disability, particularly as the life expectancy of PWH now more closely matches that of their healthy peers. Interestingly, obesity was found to be not a risk factor for CVD events in an analysis of 185 adult hemophiliacs [21]. In our hemophilia cohort, we also found that PWH with obesity did not have a greater risk for CVD events.

Aging is also a known risk factor for CVD events. Interestingly, a population-based analysis has shown that the mean age at diagnosis of CVD events among PWH was younger than that in the general population: 49.0 (95 % CI, 43.6–54.5) and 55.8 years (95 % CI, 54.5–57.0), *P* = 0.019 [18]. Although it is probably because hemophilia is a hereditary disorder which is diagnosed early with close follow-up and hence picking up CVD events, these results are suggestive of the need for earlier screening for the age-related comorbidities among PWH.

### Occurrence and survival of cancer in Hemophilia patients

Malignancies, just like CVD events, emerge as one of the important causes of morbidity and mortality in PWH nowadays [9, 28–38]. In the previous decade, most reports focused on the epidemiology and outcome of blood-borne cancers in the hemophilia patients group, as the incidence of HBV, HCV and HIV infection were high among them. In recent decades, some animal models imply that inherited coagulation disorders like hemophilia can inhibit metastasis, but substitution of coagulation factors seems to support metastasis [39]. In addition, clinical investigation has suggested the effect of hemophilia on cancer-related mortality. Since the recombinant CFCs are widely introduced, physicians are paying attention to non-virus related malignancies. However, there was limited data focusing on the epidemiology of non-virus related malignancy in PWH [9, 30]. In 2000, Soucie et al found non HIV- or liver-related cancer caused 2.2 folds of death adjusted with standard mortality rate [40]. In 2009, Miesbach W et al also found that the prevalence of cancer in elderly PWH was four times higher than in the age matched general population when hepatocellular carcinoma (HCC) was excluded [30]. However, Plug I et al found that when HCC was excluded, malignancy related death did not increase among PWH [41]. In contrast, Walker et al. reported that among HIV negative PWH in Canada, after excluding liver cancer and lymphoma, cancer related deaths were less than expected [1]. Furthermore, in a systemic review by Miesbach et al, when HIV and hepatoma were excluded, standard mortality rate of PWH are decreased [9]. Generally, the discrepancy of prevalence and outcome of cancer may be caused by conduction in different periods, which would be affected by the widespread introduction of CFCs and modern comprehensive hemophilia care. Importantly, all these studies are of insufficient sample size, and therefore carry a high risk of type 1 or 2 errors.

We conducted a nationwide and population-based analysis on the occurrence and survival of cancer in PWH between 1997 and 2010 [42]. The data in the cohort study showed that the prevalence of cancers among PWH was higher than general population (odd ratio 2.42, 95 % CI 1.74–3.35). After excluding patients with HIV or HCV infection, there was still significant difference between hemophilia and general group (odd ratio 1.66, 95 % CI 1.06–2.59). In addition, interestingly, PWH were diagnosed cancers at a much younger age. It is plausible that there shall be other causes of cancer progression among PWH. Frequent radiation exposure from diagnostic image and intraarticular therapy in PWH may play a role, as it was documented that higher radiation accumulation might increase cancer occurrence and progression [43]. This hypothesis still needs further prospective observation trials to prove. Repeated bleeding and chronic inflammation from hemophiliac arthropathy has been linked to cancer induction and metastasis [44, 45]. For PWH, this can be one important factor affecting cancer progression, but no direct link was proven yet.

**Fig. 1:**
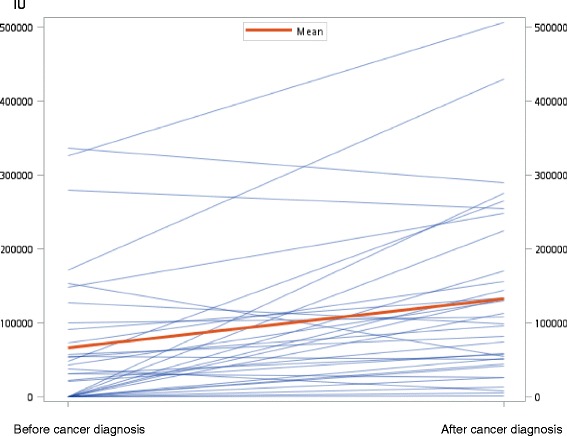
Comparison of annual amount of clotting factor used before and after cancer diagnosis among 33 hemophilia A patients (*p* = 0.0002)

In addition, the study showed that PWH had similar survival time with the general population after acquiring cancer. Factor VIII deficiency and following reduced thrombin activation in PWH can lead to reducing cancer progression in several vitro studies [46, 47]. In mice experiments, Langer R et al have proved that clotting factor VIII can enhance tumor metastasis, and reduced by a direct thrombin inhibitor [39]. It is still not known whether long-term treatment with CFCs can intensify thrombin induced metastases, or the replacement dosage is too low to generate such effect among PWH [9]. Therefore, we did further investigation on the average amount of clotting factor use before and after cancer diagnoses (Fig. 1). Among 33 patients with hemophilia A and cancer, there were 22 patients who needed replacement therapy before cancer diagnosed. The remaining 11 patients did not need replacement therapy, and started replacement afterwards. We compared the average amount of clotting factor VIII in these PWH used before and after cancer diagnosis, and found a significant increase in demand. It is plausible that therapeutic surgical procedures that warrant CFC coverage or any bleeding complications following chemotherapy requiring CFC replacement contributes to the increase in demand of CFC after cancer diagnosis. However, this leads us to consider about whether the intensity of CFC use after PWH acquiring cancer is associated with prognosis of cancer. Further research is needed to elucidate whether CFC administration will lead to cancer progression in human patients. And, a large cohort study is needed to investigate that whether large amount of excess CFC use in PWH with cancer would lead no protection of clotting deficiency from cancer treatment can lead to cancer progression.

## Conclusions

Studies on age-related comorbidities in PWH are increasingly reported. To date, there is little large-scale and prospective survey involving epidemiology of PWH with comorbidities of CVD events and cancer, and the risk for these aging diseases in PWH is often left to physician’s discretion. The current evidence showed the prevalence of CVDs among PWH seems to be comparable to that of the general population. Some known risk factors for CVD events, including aging, hypertension, and hyperlipidemia are also associated with the occurrence of CVD events in PWH. There is no evidence showing occurrence of the event directly to CFC administration. In the other hand, the incidence of non-virus related cancer seems to be higher in PWH than the general population. In addition, PWH with cancer were younger at the time of diagnosis. In regards to hemophilia effect on cancer prognosis, further basic and clinical research is needed.

## Abbreviations

CFC: Clotting factor concentrate
CVD: Cardiovascular disease
HCC: Hepatocellular carcinoma
HCV: Hepatitis C virus
HIV: Human immunodeficiency virus
NHIRD: National Health Insurance Research Database
PWH: Persons with hemophilia
